# A rapid microwave synthesis of green-emissive carbon dots with solid-state fluorescence and pH-sensitive properties

**DOI:** 10.1098/rsos.180245

**Published:** 2018-07-11

**Authors:** Tingting Yu, Haijiao Wang, Chongzheng Guo, Yanli Zhai, Jianzhou Yang, Jianhui Yuan

**Affiliations:** 1 Department of Preventive Medicine, Changzhi Medical College, Changzhi 046000, People's Republic of China; 2 Xinxiang Key Laboratory for Biomedical Materials, College of Life Science and Technology, Xinxiang Medical University, 601 Jinsui Road, Xinxiang 453003, People's Republic of China

**Keywords:** carbon dots, phthalic acid, green fluorescence, solid state, pH sensing

## Abstract

The emerging carbon quantum dots (CQDs) have been attracting significant attention for their prominent fluorescence, excellent stability and outstanding biocompatibility. Here, we report a facile one-step synthesis of highly fluorescent CQDs by using phthalic acid and triethylenediamine hexahydrate as precursors through a simple microwave-assisted method. The reaction time needed is only 60 s, which is less time-consuming than most previous reports. The phthalic acid with a benzene ring can improve the photoluminescence properties of CQDs as it can provide foreign *sp*^2^ conjugating units, and then finally result in long-wavelength emission. The synthesized CQDs were fully characterized by transmission electron microscopy, X-ray photoelectron spectroscopy and Fourier transform infrared spectroscopy. Besides, the impacts of different freed ratio on physical and chemical properties of CQDs were investigated in detail. The prepared CQDs exhibited strong green fluorescence with a broad maximum emission wavelength. The quantum yields of the CQDs can reach 16.1% in aqueous solution and they were successfully used in cell imaging with good biocompatibility. Moreover, in solid state, the CQDs with the feed ratio of 1 : 0.5 showed a strong green–yellow fluorescence which may have great potential to fabricate optoelectronic devices. Furthermore, the prepared CQDs also showed high pH sensitivity and can act as a fluorescence nanosensor for pH sensing.

## 1. Introduction

In recent years, carbon quantum dots (CQDs) have drawn tremendous attention in the nanotechnology field due to their captivating properties, such as excellent photo-stability, favourable biocompatibility and good water solubility [1,2]. These features make CQDs become new-generation fluorescent materials and to be alternatives to conventional fluorescent organic dyes and semiconductor quantum dots. Since the first report of CQDs in 2004, scientists have made great efforts to explore the synthesis methods of CQDs. Until now, a variety of CQDs have been synthesized and used in chemical sensing, bio-sensing, bio-imaging, nanomedicine, photocatalysis and electrocatalysis [3,4]. Among these reports, the synthesis methods can be mainly divided into two categories: top-down and bottom-up routes. The former breaks bulk carbon materials, such as nanodiamonds, graphite, carbon nanotubes, carbon soot, activated carbon and graphite oxide into small pieces by the physical or chemical methods like arc discharge, laser ablation and electrochemical oxidation [1], while in the latter approaches synthesized CQDs are mainly from the elaborately selected molecular precursors (such as citrate, carbohydrates, glucose, l-glutamic acid and ethanol) through combustion, thermal treatments or simple microwave (MW)-assisted routes. Obviously, the bottom-up synthesis routes have many advantages, such as simple synthetic conditions, precursors are cheap and easy to obtain, short reaction times, etc. [5]. Thus, solvothermal method and MW-assisted method as two very simple bottom-up methods for the synthesis of CQDs have been widely used.

In the process of chemical synthesis, short reaction time has been the goal that scientists have been trying to achieve. Among the above two synthetic methods, solvothermal method often requires higher reaction temperature (greater than 150°C) and longer reaction time (greater than 0.5 h) [6]. However, MW-assisted method only takes a few minutes and the synthetic devices are also very simple. So far, large amount of CQDs were synthesized by MW-assisted method with a very short time. Such as Dong and co-workers [7] used chitosan, ethanolamine and acetic acid as precursors through 15 min MW-assisted pyrolysis process to prepare CQDs for the detection of arginine and Cu^2+^. Lin and co-workers [8] used citric acid and amino compound-containing hydroxyls (such as ethanolamine and tris(hydroxylmethyl)aminomethane) as precursors through 5 min MW-assisted pyrolysis process to prepare the CQDs with ultra-high fluorescence quantum yields. Wang and co-workers [9] prepared nitrogen and phosphorus co-doped carbon dots (N, P–C-dots) for fluorescent cell imaging by 7 min MW-assisted thermolysis of *N*-phosphonomethyl aminodiacetic acid and ethylenediamine. Until now, a variety of CQDs have been synthesized and used in chemical sensing, bio-sensing, bio-imaging, nanomedicine, photocatalysis and electrocatalysis. However, there are still some drawbacks that limit the applications of CQDs, such as their inability to emit strong long-wavelength fluorescence and aggregation-caused quenching [10,11].

In this work, we report the preparation of CQDs by a facile, green and less time-consuming MW-assisted method using phthalic acid and triethylenediamine hexahydrate as the precursor with two feed ratios. The reaction time needed is only 60 s, which is less time-consuming than most previous reports. The effect of feed ratio on physical and chemical properties of CQDs was investigated in detail. The prepared CQDs can emit strong green fluorescence with about 16% quantum yield and successfully used for cell imaging with good biocompatibility. Moreover, the CQDs also show good pH sensitivity and can be used as pH nanosensor. Furthermore, to our delight, the CQDs also can emit strong yellow–green fluorescence in solid state at the feed ratio of 1 : 0.5, which was seldom reported and may have great potential to produce optoelectronic devices such as light-emitting diodes.

## 2. Experimental

### 2.1. Materials and measurements

Phthalic acid and triethylenediamine hexahydrate were obtained from Sigma–Aldrich and used as received. All reagents were used as received without further purification. Deionized water was used throughout the experiment. Fourier transform infrared spectroscopy (FT-IR) was performed on an FT-IR Nicolet 380 spectrometer. Transmission electron microscopy (TEM) observations were performed on Tecnai G2 F20 S-TWIN. Fluorescence emission spectra were obtained using a FluoroMax-4 Spectrofluorophotometer (HORIBA JobinYvon) at 298 K. The time-resolved fluorospectroscopy was performed using an FLS 920 spectrometer. HeLa cells lines were purchased from Shanghai Institute of Biochemistry and Cell Biology, Chinese Academy of Sciences.

### 2.2. Preparation of carbon quantum dots

Briefly, phthalic acid (2 g) and triethylenediamine hexahydrate with different feed ratio (w/w = 1 : 0.5, 1 : 1, 1 : 2, 1 : 3) were dissolved in 3 ml deionized water in the 100 ml beaker. Then the beaker was placed at the centre of the rotation plate of a domestic MW oven (700 W) and heated for 60 s. After cooling down, the crude products were dialysed against 500 ml of deionized water for 24 h and lyophilized to yield powdered CQDs

### 2.3. Cytotoxicity assays

Toxicity was determined by CellTiter 96®AQ_ueous_ One Solution Cell Proliferation Assay. About 10 000 cells per well were seeded into 96-well plates and cultured for 24 h. Then the medium was exchanged with a serum containing culture medium (100 µl) containing different amounts of CQD. The cells were further incubated for 24 h. After that, the solutions were removed, 100 µl PBS containing 20 µl CellTiter 96®AQ_ueous_ One Solution Cell Proliferation was added to each well for additional 1 h incubation at 37°C. Then, the absorbance of each sample was measured using an ELISA plate reader (model 680, BioRad) at a wavelength of 490 nm. The cell survival was expressed as follows: cell viability = (OD_treated_/OD_control_) × 100%.

### 2.4 Confocal laser scanning microscopy analysis

HeLa cells were seeded in a 35 mm confocal dish (Φ = 15 mm) at a density of 2 × 10^4^ cells per well. Under standard incubation conditions, the medium was exchanged with serum-containing medium after 24 h. Then cells were incubated with CQDs in media for 24 h at 37°C. After incubation, cells were rinsed twice with PBS (pH 7.4). The confocal laser scanning microscopy (CLSM) observation was performed using Leica TCS SP5 at excitation wavelengths of 405 nm.

## 3. Results and discussion

### 3.1. Physicochemical characterization of carbon quantum dots

**Figure 1.**
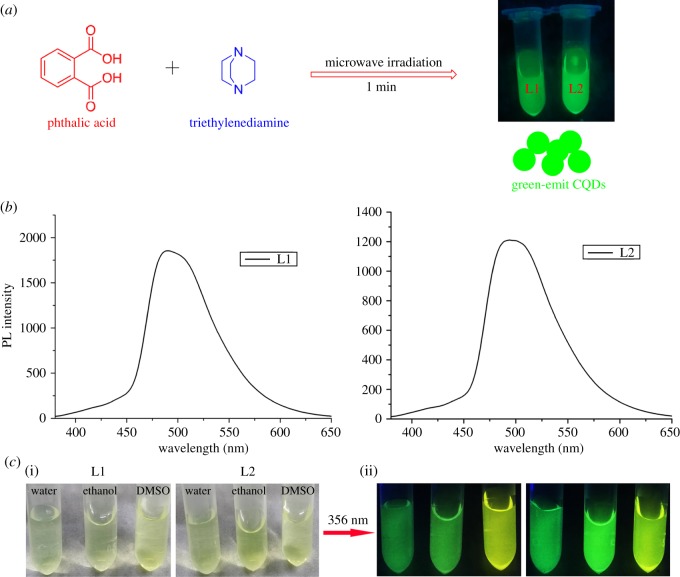
(*a*) Schematic illustration for the synthesis of the CQDs. (*b*) The corresponding PL spectra of the CQDs. (*c*) Photographs of the CQDs in different solution under daylight (i) and fluorescence images (ii) under UV light (excited at 365 nm).

In this work, CQDs were prepared by a facile one-step MW-assisted pyrolysis of phthalic acid and triethylenediamine hexahydrate with different feed ratio. The CQDs synthesized at the feed ratio of 1 : 0.5, named as L1, and at a feed ratio of 1 : 1, named as L2. As shown in figure 1, the reaction time of MW-assisted pyrolysis process only needs to be 60 s, indicating the fast carbonation and restructuring process of phthalic acid and triethylenediamine hexahydrate. The prepared CQDs can emit strong green fluorescence in aqueous solution instead of the common blue fluorescence under UV light (365 nm, figure 1*c*). So far, the PL mechanism for CQDs still remains unclear, especially the long-wavelength emission. Some reports ascribed the PL red shift of CQDs to conjugated *sp*^2^-domain effect [12–15]. Meanwhile, using the precursors with big polyaromatic structures to synthesize CQDs seems to be more likely to obtain fluorescence with long-wavelength emission in previous report [14]. Therefore, we used the phthalic acid with a benzene ring as carbon source and expected it can help the CQDs to improve the fluorescence wavelengths with red shift. On the other hand, cyclic amine is seldom used as a passivant. The triethylenediamine hexahydrate with cyclic structure used as the passivant was expected to provide some special optical properties and improve the quantum yield of CQDs. In addition, the prepared CQDs also show solubility in polar organic solvents such as DMSO and ethanol, and can emit yellow–green and green colour fluorescence under UV light (365 nm, figure 1*c*). Meanwhile, the photoluminescence emission (PL) spectra of the CQDs were also evaluated at 365 nm. Unlike other reports, both L1 and L2 showed a broad emission peak with a maximum emission wavelength in the range of 480–510 nm. The Commission Internationale de L'Eclairage (CIE) coordinates of L1 and L2 from their PL spectra exhibited green colour with CIE coordinates of (0.18, 0.43) and (0.19, 0.45), respectively, which were consistent with the intuitive colour (electronic supplementary material, figure S1).

**Figure 2.**
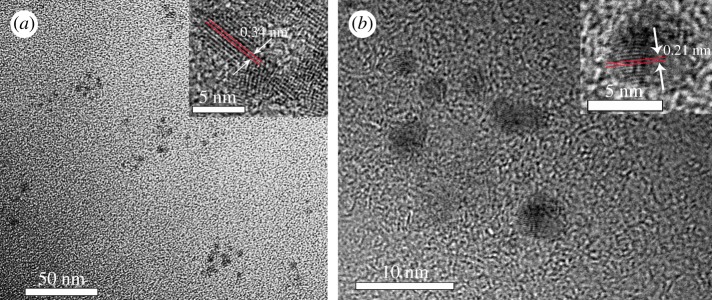
TEM and HRTEM (inset) images of (*a*) L1 and (*b*) L2.

**Figure 3.**
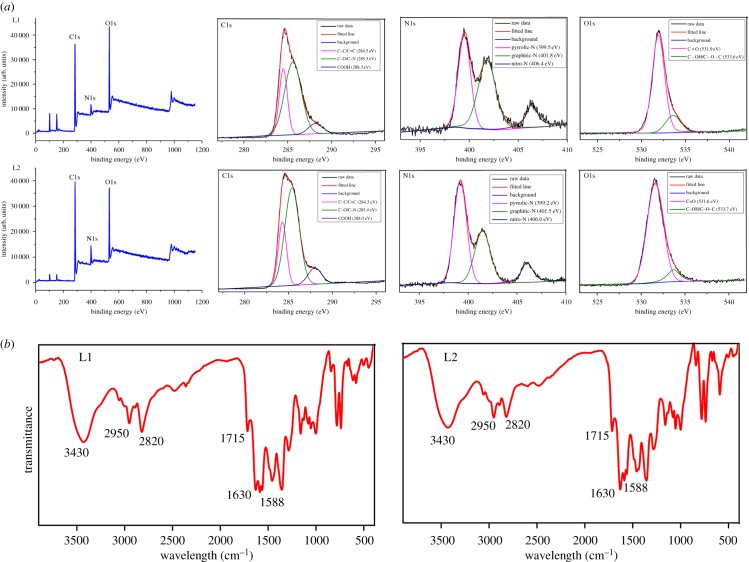
(*a*) XPS spectra of the CQDs and (*b*) FT-IR spectra of CQDs.

TEM (figure 2) demonstrate that all the as-prepared CQDs are monodispersed with similar sizes in the range of 2–6 nm and their mean particle diameters were measured to be 3.5 ± 0.7 and 3.7 ± 0.8 nm for L1 and L2, respectively (electronic supplementary material, figure S2). The high-resolution TEM (HRTEM) micrographs of CQDs (figure 2, inset) exhibit well-resolved lattice fringes with a typical d-spacing of 0.34 nm for L1 and 0.21 nm for L2, which correspond to the (100) and (002) in-plane lattice of graphene, respectively [16–18]. The results suggest that the different feed ratio of the CQDs impact their lattice spacing. The chemical composition and structure of L1 and L2 were further investigated by X-ray photoelectron spectroscopy (XPS). Both L1 and L2 show three prominent peaks of C1s at 285 eV, N1s at 400 eV and O1s at 532 eV [19,20]. In the high-resolution spectra (figure 3*a*), the C1s spectrum of both L1 and L2 can be resolved into three peaks at 284.5, 285.6 and 288.3 eV, attributed to C─C/C═C, C─O/C─N and COOH groups, respectively [21,22]. N1s spectrum for L1 and L2 also can be resolved into three peaks at 399.5, 401.8 and 406.4 eV, which should be attributed to the presence of pyrrolic-N, graphitic-N and nitro-N, respectively [23]. The O1s spectrum exhibits two oxygen states of C═O at 531.9 eV and C─OH/C─O─C at 533.6 eV, respectively [24]. Furthermore, the elemental content ratio of C, N and O are also calculated (electronic supplementary material, table S1). Obviously, L2 has higher nitrogen content, which is consistent with their feed ratio. FT-IR was further employed to identify the organic functional groups on L1 and L2 (figure 3*b*). Both L1 and L2 exhibit a broad peak from 3100 to 3700 cm^−1^ corresponding to O─H and N─H stretching vibrations, indicating the presence of carboxylic acid and amino groups [25]. The amide bond is also confirmed by the typical peaks at 1630 cm^−1^, attributed to the vibrations of amide's C═O, indicating successful conjugation between amines of triethylenediamine hexahydrate and carboxyls of phthalic acid. The peaks at 1715 and 1588 cm^−1^ are attributed to stretching vibrations of carboxyl's C═O and C═C, respectively. Finally, the typical peaks at 2920 and 2850 cm^−1^ are attributed to stretching vibration of methylene, which may be derived from triethylenediamine hexahydrate. Obviously, the XPS and FT-IR data conclude that L1 and L2 have the similar functional groups and the different feed ratio does not influence the types of their functional groups.

**Figure 4.**
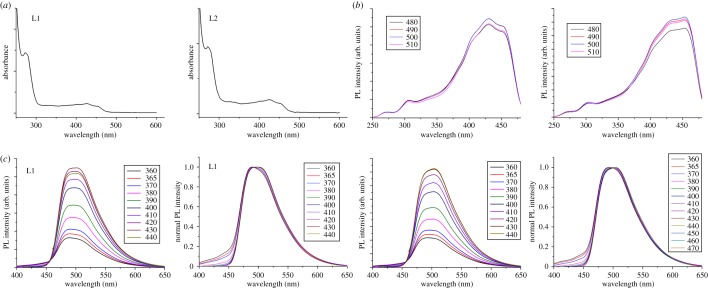
(*a*) UV–Vis absorption (Abs) of the CQDs. (*b*) Fluorescence excitation (Ex) spectra of the CQDs. (*c*) Excitation-dependent and normalized PL spectra of the CQDs.

To further explore the optical properties of the CQDs, UV–Vis absorption is researched at room temperature. As shown in figure 4*a*, the UV–Vis spectrum of the aqueous solution exhibits a sharp peak at 275 nm and a broad absorption band with peaks in a range of 350–480 nm, respectively. The peak at 275 nm, attributed to π−π* transitions of carbon core C═C units, leads to nearly no observed PL signal, while the latter broad absorption due to the trapping of excited states results in strong emission [26,27]. Furthermore, the excitation spectrum of the CQDs were also measured (figure 4*b*). Both L1 and L2 have broad excitation peaks in a range of 290–480 nm, which well matched the UV–Vis absorption spectrum. The maximum excitation wavelengths, of L1 and L2 are about 430 and 454 nm, respectively. In addition, at different emission wavelength (*λ*_em_), their excitation spectra nearly have no changes. As depicted in figure 4*c*, the fluorescence intensity increases remarkably with excitation wavelength from 360 to 440 nm. Meanwhile, the normalized photoluminescence spectra revealed a small emission peak shift in the excitation wavelength range of 360–440 nm (Δ*λ*_em_ = 10 nm, figure 4*c*), which is in agreement with previous literature [28,29]. By using quinine sulfate in 0.10 M of H_2_SO_4_ (quantum yield 54%) as standard sample, the quantum yield of the prepared CQDs are calculated to be 15.8% and 16.1%, respectively, which is higher than other reports [30–32]. It seems that the amount of passivator does not have a significant influence on the fluorescence properties of such CQDs. The report has found that the PL lifetime of CQDs may be inequable at different excitation wavelength [33]. Then the time-resolved PL spectrum of the CQDs was measured in solution and the PL decay curves of L1 and L2 are well fitted by a mono-exponential formula (electronic supplementary material, figure S3). The PL lifetimes of L1 excited at 365, 390 and 440 nm are 4.6, 4.7 and 4.9 ns, respectively, and for L2 are 5.0, 5.0 and 5.1 ns, respectively. The results showed L1 and L2 have almost the same PL lifetime at different excitation wavelength. So far, the PL mechanisms of CQD can be divided into two categories: one is based on the band gap transitions in conjugated π-domains, which originate from the quantum confinement effect, and the other is related to surface defects on the CQDs [34,35]. As the maximum excitation wavelength agrees well with the corresponding absorption peak (figure 4) and considering the above PL property of the CQDs, it may indicate that the emission is characterized by band-edge exciton-state decay rather than defect-state decay [35–37]. On the other hand, like Chi and co-workers [12] used rhodamine B as the foreign *sp*^2^ conjugating units to prepare tunable long-wavelength emission CQDs, we believed the phthalic acid with a benzene ring also can serve as foreign *sp*^2^ conjugating units and big polyaromatic structures to construct CQDs with long-wavelength emission.

### 3.2. pH sensing

**Figure 5.**
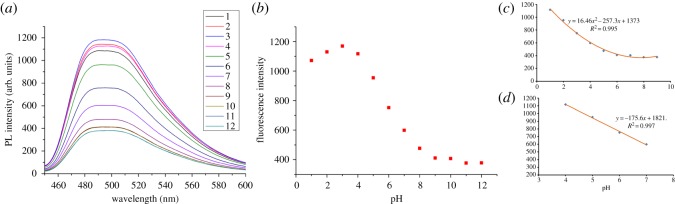
(*a*) Fluorescence emission spectra of L2 in 10 mM phosphate-buffered solutions at various pH values. (*b*–*d*) Plots of F504 versus pH. F504 stands for the fluorescence intensities at 504 nm.

In recent decades, pH sensing in aqueous media has attracted a great deal of interest considering the important role of pH in a broad range of applications from environmental to industrial to biomedical systems, such as analytical chemistry, cellular biology, medicine and environmental protection [5]. Therefore, these prepared CQDs were tried for pH sensing in aqueous. To our delight, the fluorescence intensity of the both L1 and L2 were sensitive to pH in a broad range of 1–12. As shown in figure 5 and electronic supplementary material, figure S4, with the pH increase, the fluorescence intensity of L1 and L2 firstly increased and then the decreased remarkably. When the fluorescence intensity of L2 at 504 nm was plotted as a function of pH (3–12), a well-fitted curve was obtained using the polynomial function and as shown in figure 5*b–d*. Moreover, under the pH range of 3–6, the fluorescent emission intensity showed good linearity with pH variation (figure 5*d*). These fitting curves were calculated to be *y* = 16.46 *x*^2^ + 257.3*x* + 1317 with *R*^2^ = 0.995 and *y* = −157.6*x* + 1821 with *R*^2^ = 0.997, respectively. Thus, the as-synthesized carbon dots can be explored as a potential pH nanosensor for biological systems.

### 3.3. Solid-state fluorescence of the carbon quantum dots

**Figure 6.**
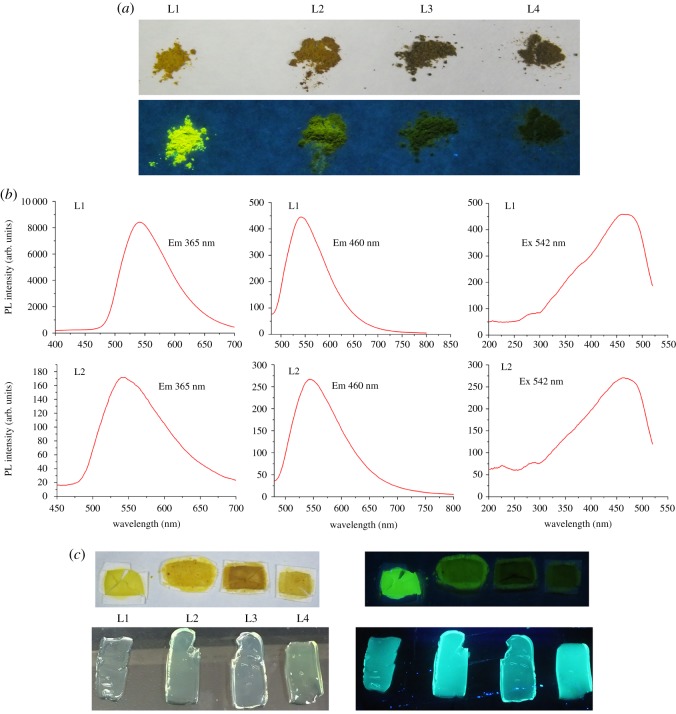
(*a*) Photographs of the CQDs under daylight and irradiation at 365 nm. (*b*) SSF spectra of the CQDs. (*c*) The KBr infrared tablet and agarose gel photographs of the CQDs under daylight and irradiation at 365 nm.

To date, almost all CQD-related reports have concentrated on homologous aqueous solution fluorescence due to CQDs always undergo self-quenching in the solid state, like organic molecules ascribed to excessive resonance energy transfer or direct π–π interactions. Only few reports have just mentioned solid-state fluorescence (SSF) of CQD powder and the SSF is much beneficial to numerous applications, such as optoelectronic devices and sensors, which generally require photoluminescent materials emitting in the solid state [38]. Interestingly, though L1 and L2 have a similar composition, the prepared L1 can emit strong green–yellow SSF (figure 6*a*) under 365 nm, while L2 only emits very weak SSF. Then the CQDs with feed ratio of 1 : 2 and 1 : 3 (named as L3 and L4) were also prepared and they showed no SSF. In addition, when the reaction time was increased to 2 min for L1 and L2 (named as L1–2, L2–2, respectively), the results showed that L1–2 and L2–2 still emit strong green fluorescence in aqueous solution (electronic supplementary material, figure S5*a*). However, their SSF became very weak (electronic supplementary material, figure S5*b*). L1–2 only showed a faint green–yellow fluorescence in solid state and the SSF of L2–2 even disappeared. The SSF mechanism of these CQDs is poorly understood; however, these phenomena indicated that feed ratio of reactants and the reaction time may be the key factors which can seriously affect the SSF of CQDs, and the SSF of this type CQDs can only be obtained at a suitable feed ratio and reaction time. The emission spectrum and excitation spectrum of L1 and L2 were also measured; both of them showed a maximum emission peak at 542 and 520 nm, respectively (figure 6*b*). In order to observe their fluorescence in more detail, the KBr infrared tablet and agarose gel which contained L1, L2, L3 and L4 powder were made. Obviously, only L1-derived KBr infrared tablet can emit strong fluorescence, while for the agarose gel, all of them can emit strong fluorescence (figure 6*c*). These results demonstrated that our prepared CQDs have broad application prospect and the SSF of L1 may have a potential for fabricating optoelectronic devices.

### 3.4. Cell imaging

The cell imaging ability of the prepared CQDs was also investigated. Firstly, the cell viability after 24 h incubation with CQDs was evaluated before using the L1 and L2 for the cell imaging (electronic supplementary material, figure S6). As expected, L1 and L2 only showed negligible cytotoxicity under the measured range, indicating their good biocompatibility. To further evaluate the bio-imaging ability of L1 and L2, both of them were incubated with HeLa cells, and observed by CLSM. As shown in electronic supplementary material, figure S7, green fluorescence could be found in HeLa cells owing to the strong fluorescence emitting from L1 and L2. Under the excitation wavelength of 405 nm, the CQDs could stain cells with green colour, and this further confirmed the great potential of the CQDs in serving as optical nanoprobes for bio-imaging applications.

## 4. Conclusion

A one-pot method has been proposed to synthesize long-wavelength emissive CQDs with SSF properties and pH sensitivity via a facile, green and less time-consuming simple MW-assisted method. The phthalic acid used in the experiment offered the foreign *sp*^2^ conjugating units for the CQDs and finally resulted in the long-wavelength emission property. The results showed CQDs could be formed in just 60 s and emit strong green fluorescence with about 16% quantum yield. Then the synthesized CQDs were fully characterized by TEM, XPS and FT-IR. TEM indicates all the as-prepared CQDs are monodispersed with similar size distribution in the range of 2–6 nm. Their mean particle diameter was measured to be 3.5 and 3.7 nm for L1 and L2, respectively. Meanwhile, different lattice spacing was also obtained by the different freed ratio. The FT-IR and XPS suggested that L1 and L2 have the similar composition. Both L1 and L2 exhibit excellently sensitive to pH in a broad range as well as act as cell-imaging reagents with good biocompatibility. Interestingly, the obtained L1 not only can emit green fluorescence in aqueous solution, but also can emit strong green–yellow in solid state which is much beneficial for many applications, such as optoelectronic devices and sensors. In short, these results demonstrated the prepared CQDs are multi-functionals and may have the application potential in the field of optoelectronic devices, sensors and bio-imaging.

## Supplementary Material

supplementary material

## Data accessibility

Electronic supplementary material can be found online on the website of the publisher.
