# Supplementary material for: A rapid microwave synthesis of green-emissive carbon dots with solid-state fluorescence and pH-sensitive properties
Source: R Soc Open Sci. 2018 Jul 11;5(7):180245. doi: 10.1098/rsos.180245 (PMC6083653; doi:10.1098/rsos.180245)
Supplement: supplementary material [file rsos180245supp1.doc]

**Supplementary material**

**A rapid microwave synthesis of green-emissive carbon dots with** **Solid-State Fluorescence and pH-sensitive properties**

Tingting Yu^1, †^, Haijiao Wang, ^2^, ^†, *^, Chongzheng Guo^1^, Yanli Zhai ^1^, Jianzhou Yang^1^ and Jianhui Yuan ^1^

^1^ Department of Preventive Medicine, Chang Zhi Medical College, Changzhi 046000, China.

^2^ Xinxiang Key Laboratory for biomedical materials, College of Life Science and Technology, Xinxiang Medical University, 601 Jinsui Road, Xinxiang 453003, China

* Corresponding authors: [taiyiyibo@126.com](mailto:taiyiyibo@126.com) (H. Wang); Tel.: +86-0355-3151068

† These authors contributed equally to this work.

**Table S1. The C, N and O content ratio of CQDs**.

|  | C : N : O |
| --- | --- |
| **L1** | 47.8 : 8.5 : 43.7 |
| **L2** | 50.1: 13 : 36.9 |

**
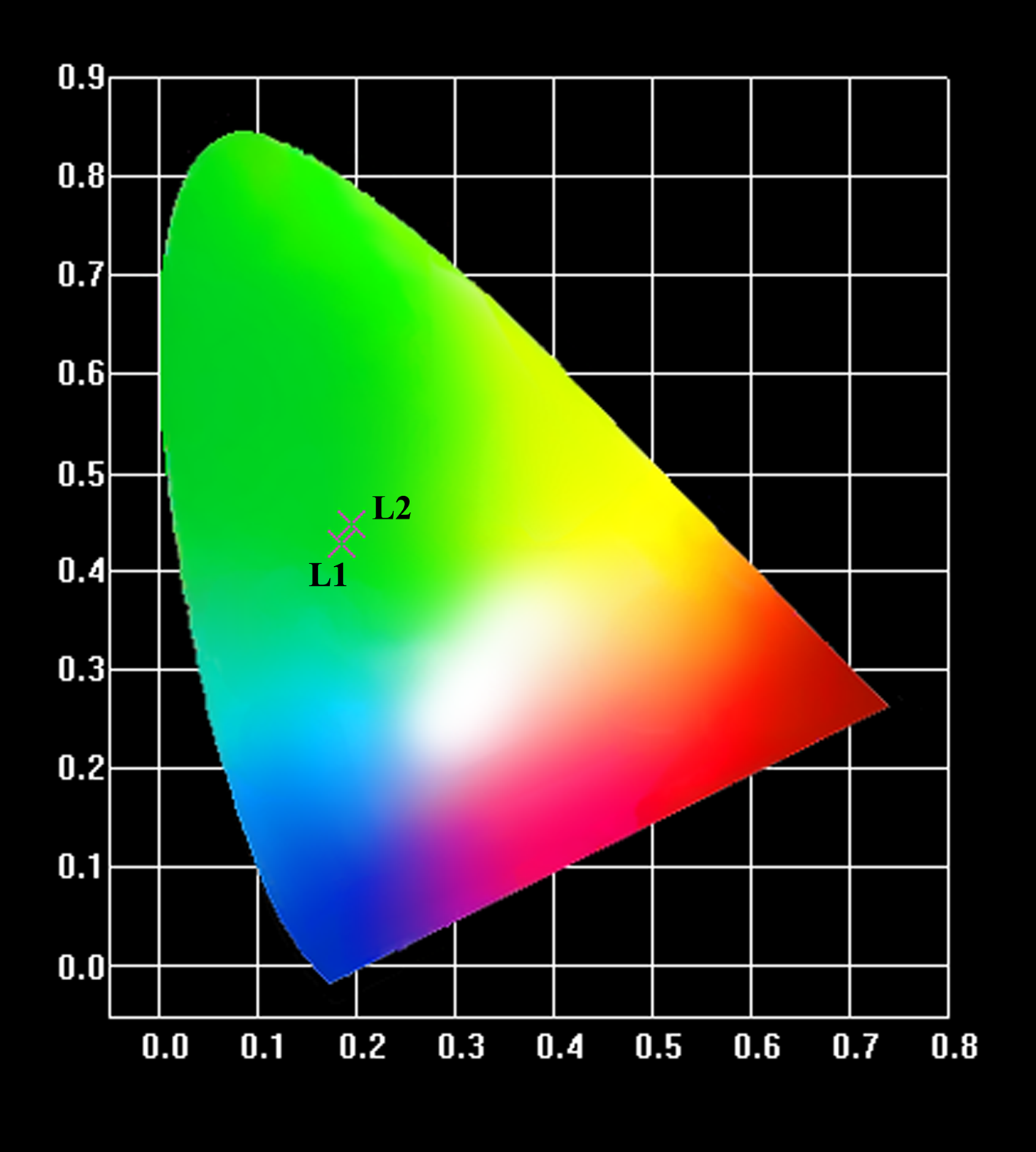
**

**Figure S1.** CIE coordinates of the CQDs.

**
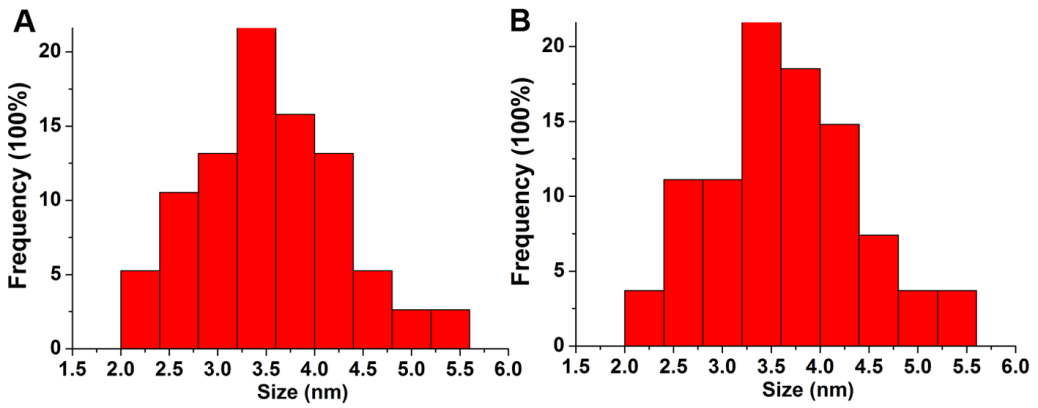
**

**Figure S2.** (A) Particle size distribution of L1. (B) Particle size distribution of L2.

**
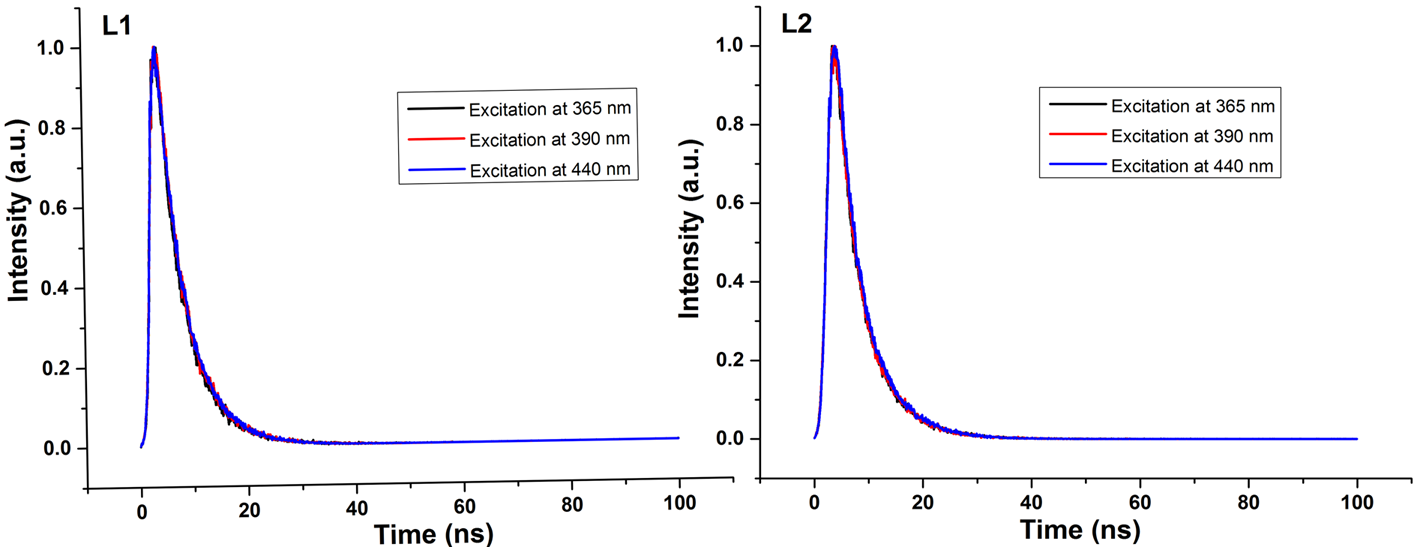
**

**Figure S3.** The ﬂuorescence-decay curves of the CQDs (emission wavelength was centred at 500 nm)

**
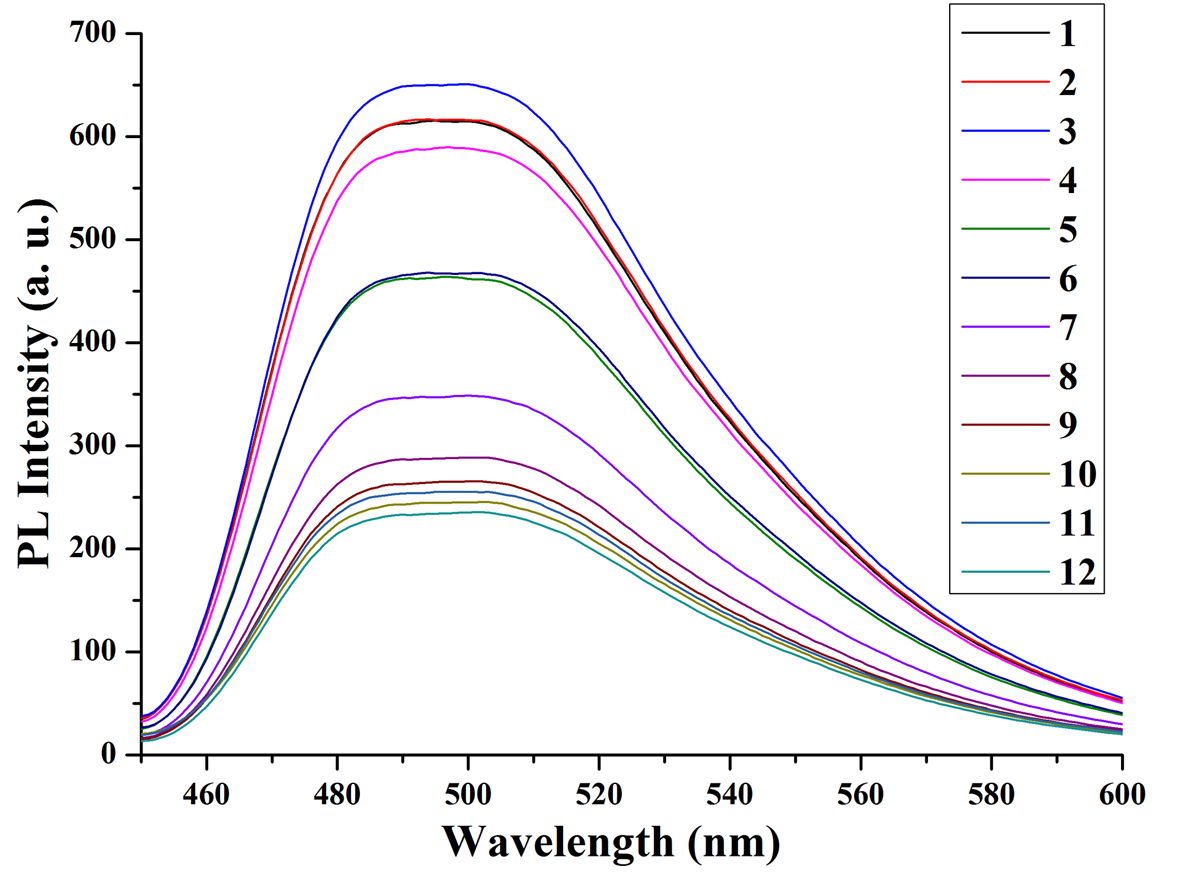
**

**Figure S4.** Fluorescence emission spectra of L1 in 10 mM phosphate-buffered solutions at various pH values.


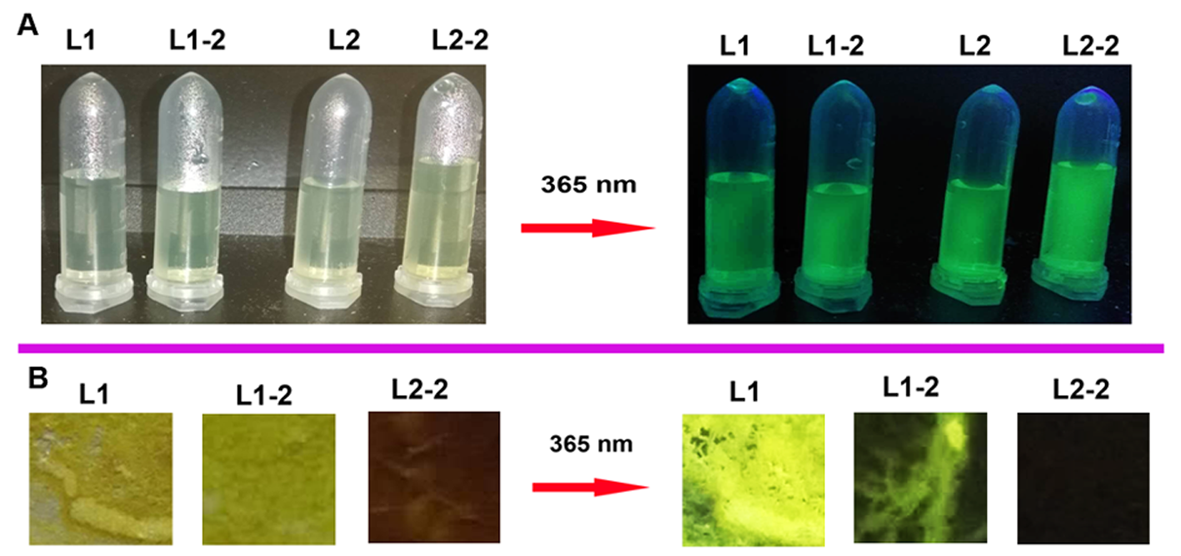


**Figure S5.** (A) The fluorescence of CQDs in aqueous solution. (B) The fluorescence of CQDs in solid state.

**
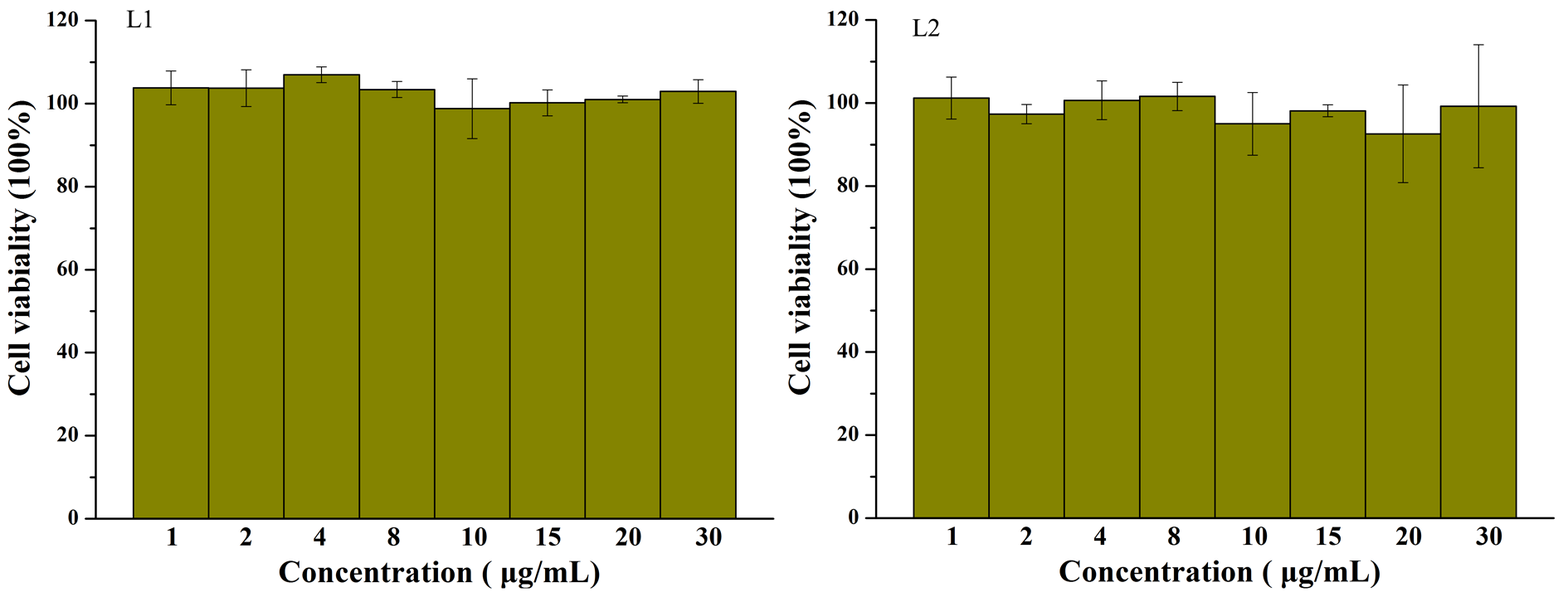
**

**Figure S6.** Cytotoxicity of CQDs at different concentration toward HeLa cells.

**
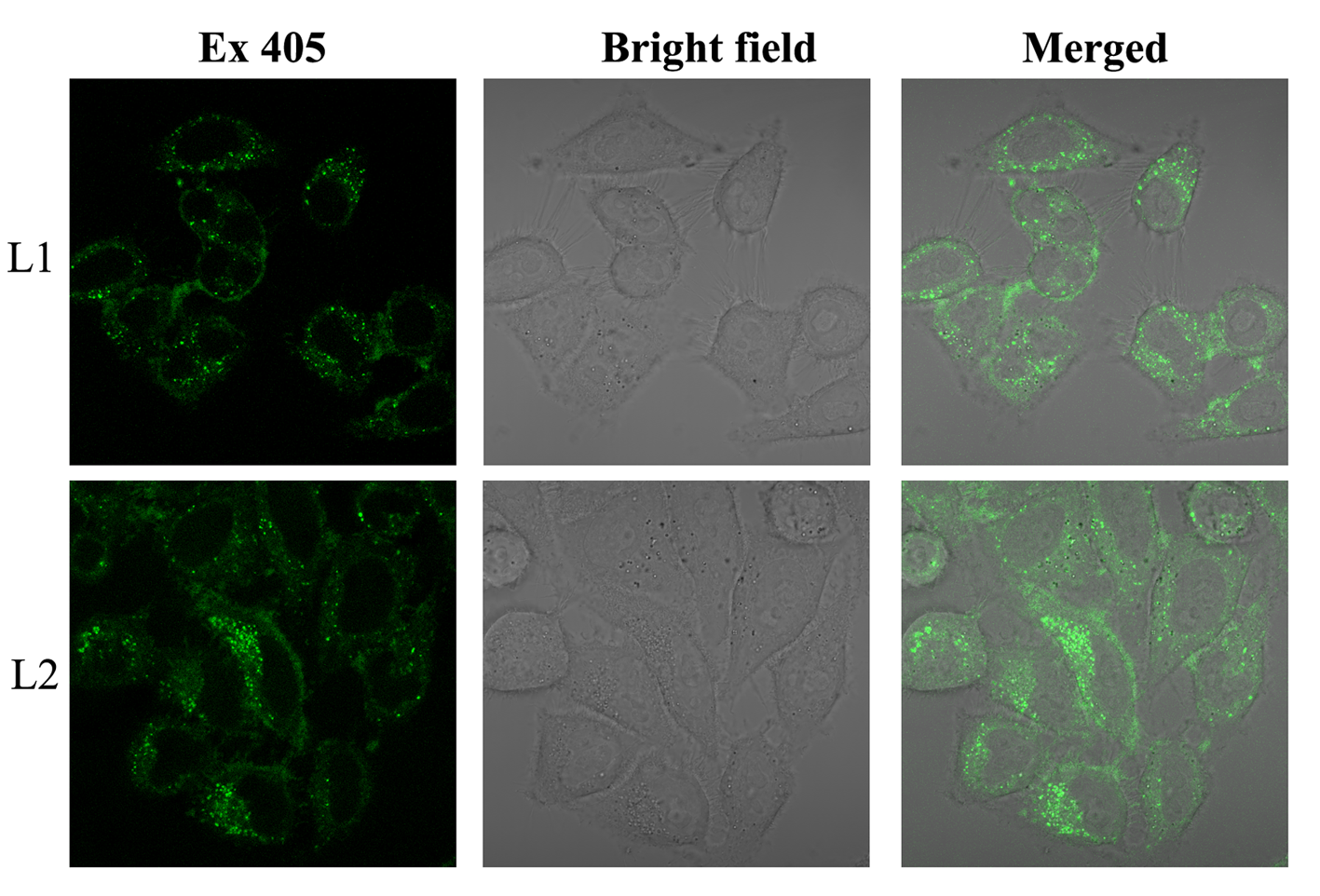
**

**Figure S7.** Representative fluorescence microscopic images of Hela cells incubated with 5 µg mL^−1^ of L1 and L2.
